# Association between carotenoid intake and metabolic dysfunction-associated fatty liver disease among US adults: A cross-sectional study

**DOI:** 10.1097/MD.0000000000036658

**Published:** 2023-12-22

**Authors:** Hang Zhang, Li Li, Lei Jia, Jinchun Liu

**Affiliations:** a First Clinical Medical College, Shanxi Medical University, Taiyuan, China; b Intensive Care Unit, The Second Hospital of Tianjin Medical University, Tianjin, China; c Institute of Hematology and Blood Diseases Hospital, Chinese Academy of Medical Sciences and Peking Union Medical College, Tianjin, China; d Department of Gastroenterology, The First Hospital of Shanxi Medical University, Taiyuan, China.

**Keywords:** carotenoids, MAFLD, lycopene, hepatic steatosis, NHANES

## Abstract

Carotenoids have been recognized for their potential health benefits due to their antioxidant properties. There is limited research on the association between metabolic dysfunction-associated fatty liver disease (MAFLD) and carotenoids. This study aimed to investigate the effect of carotenoid intake on the risk of MAFLD. We retrospectively analyzed 2722 adults aged ≥ 18 from the National Health and Nutrition Examination Survey 2017-2018. Hepatic steatosis was identified by elastography, and carotenoid consumption was evaluated through two 24-hour dietary recall interviews. Weighted logistic regression models, subgroup analyses, and restricted cubic splines were used for analyses. The weighted prevalence of MAFLD was 51.90%. Weighted logistic regression analysis demonstrated that intake of β-carotene, lutein/zeaxanthin, and lycopene was associated with a lower risk of MAFLD after adjusting for various covariates. Compared to the lowest tertile, a significant inverse correlation was observed between the highest total lycopene intake and MAFLD among females in the gender subgroup analysis. Restricted cubic spline regression analysis revealed a U-shaped association between lycopene consumption and MAFLD risk (*P* < .001), with an inflection point of approximately 9.48 mg/day. Moreover, the nonlinear relationship was particularly significant in females and absent in males. In summary, increased β-carotene, lutein/zeaxanthin, and lycopene consumption was associated with a decreased risk of MAFLD. The relationship between total lycopene intake and MAFLD was nonlinear, primarily in females. These findings have significant implications for the potential prevention and management of MAFLD.

## 1. Introduction

Metabolic dysfunction-associated fatty liver disease (MAFLD), a newly proposed definition to replace nonalcoholic fatty liver disease (NAFLD), serves as a “positive criteria” independent of excessive alcohol consumption or other concomitant liver diseases and is correlated with the patient’s metabolic risk profile.^[1]^ As a multi-systemic disease involving obesity, diabetes, and metabolic syndrome, MAFLD affects approximately 39% of the population, and the risk of advanced liver fibrosis in MAFLD patients is as high as 7.4%, making it an increasingly prominent cause of chronic liver failure requiring liver transplantation worldwide.^[2,3]^ People living with MAFLD also have higher all-cause, cardiovascular-related, and other-cause mortality based on the findings of the Third National Health and Nutrition Examination Survey (NHANES III), while currently having no approved specific pharmaceutical treatments.^[4]^ Therefore, identifying modifiable risk factors and implementing effective interventions are crucial because of the growing economic and health burden of MAFLD.

Carotenoids, primarily diet-derived micronutrients, have potential health benefits in reducing the risk of certain cancers, cardiovascular diseases, and macular degeneration.^[5–7]^ Individuals with high-level carotenoids also experience a lower incidence of chronic obstructive pulmonary disease, all-cause dementia, and chronic kidney disease among American adults.^[8–10]^ According to research conducted through the NHANES 2003 to 2014, the risk of NAFLD decreased with increasing intake of carotenoids, including α-carotene, β-carotene, β-cryptoxanthin, lutein/zeaxanthin, and lycopene.^[11]^ As natural antioxidants, the consumption of β-carotene and lutein/zeaxanthin was found to be inversely correlated with liver steatosis.^[12]^ In addition, lycopene has been shown to ameliorate lipid accumulation and hepatocyte steatosis in both vivo and vitro models of NAFLD, which may be related to the “multiple-hit” hypothesis embracing diverse processes, such as insulin resistance, lipotoxicity, inflammation, and cytokines imbalance, among others.^[13]^ However, limited research exists on the association between carotenoids and MAFLD.

The objective of this study was to investigate whether carotenoid consumption could serve as a preventive factor against MAFLD, which highlights metabolic dysfunction as the core of the disease.^[14]^ For this purpose, we mainly conducted logistic regression analysis and restricted cubic spline analysis to ascertain and visualize the association between carotenoid intake and the risk of MAFLD among American adults by utilizing the NHANES database, thereby proposing novel insights into the prevention and management of MAFLD.

## 2. Materials and Methods

### 2.1. Study design and participants

The NHANES program is carried out by the National Centre for Health Statistics to monitor the nutrition and health status of American adults and children by examining a nationwide representational sample of around 5000 individuals annually.^[15]^ This cross-sectional study extracted data from the NHANES 2017 to 2018 cycle, which was the first to use FibroScan® for ultrasound and vibration-controlled transient elastography (VCTE) to obtain liver stiffness measurements and controlled attenuation parameters (CAPs) as indicators for evaluating liver fibrosis and hepatic steatosis, respectively.

Elastography measurements were performed at the NHANES mobile examination center (MEC) by a trained healthcare technician using the FibroScan® model 502 V2 Touch, which was equipped with either a medium or extra-large wand (probe). The validity of the VCTE examination requires 3 essential requirements: a minimum fasting duration of 3 hours, at least 10 complete stiffness (E) measurements, and a liver stiffness interquartile range/median E < 30%. Elastography is a noninvasive diagnostic technology that has been shown to reflect liver fibrosis (sensitivity, 93.7%; specificity, 91.1%) and hepatic steatosis (with an AUROC of 0.96).^[16–18]^ Additional details on the protocols for quality control and assurance during the VCTE procedures can be found in publicly accessible resources.^[19]^

**Figure 1.**
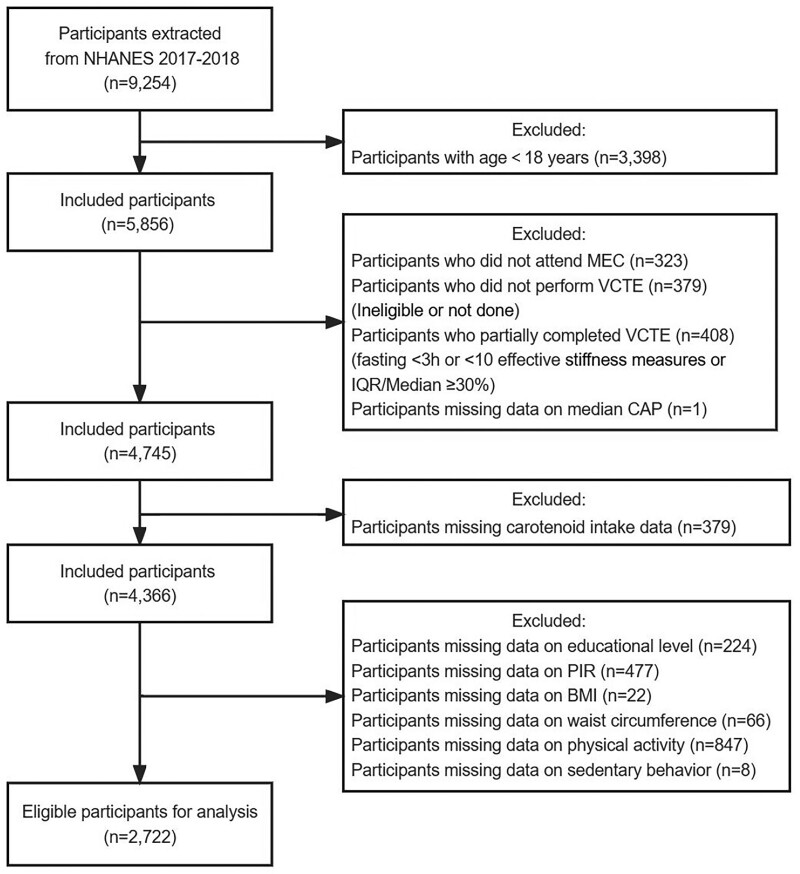
Flowchart of the sample selection from NHANES 2017–2018. BMI = body mass index, CAP = controlled attenuation parameter, IQR = interquartile range, NHANES = National Health and Nutrition Examination Survey, MEC = mobile examination center, VCTE = vibration-controlled transient elastography, PIR = poverty-income ratio.

Of the 9254 participants in NHANES 2017 to 2018, 5856 aged ≥ 18 years initially enrolled were required to attend the MEC, but 323 only underwent interviews, 379 did not perform the exam (ineligible or not done), 408 partially completed (fasting < 3 hours or < 10 effective E measures or interquartile range/Median ≥ 30%) and 1 lacked the median CAP value. Furthermore, 379 individuals missing data on dietary carotenoid intake were excluded. After further excluding individuals with insufficient data on other covariates, 2722 participants with complete information about sociodemographic data, physiological measurements, carotenoid intake, and comorbidities (hypertension, diabetes mellitus, and metabolic syndrome) remained in the final analysis, comprising 1476 individuals with MAFLD and 1246 without (Fig. 1). All participants provided informed consent for this study, which was authorized by the National Centre for Health Statistics Research Ethics Review Board (Protocol number: 2018-01) before enrollment.

### 2.2. Definition of MAFLD

Under the new definition proposed by the 2020 international expert consensus statement,^[1]^ the diagnostic criteria for MAFLD require the presence of hepatic steatosis detected through imaging techniques, blood biomarkers, or liver biopsy, in conjunction with at least one of the following 3 criteria: overweight/obesity, type 2 diabetes mellitus, or metabolic dysregulation. This study utilized an optimal CAP cutoff of 248 dB/m to identify hepatic steatosis, achieving a sensitivity of 68.8%, specificity of 82.2%, and a maximum Youden index.^[20]^ MAFLD status was defined as non-MAFLD or MAFLD based on the median CAP by VCTE measurements.

### 2.3. Carotenoid intake assessment

All NHANES participants were available to provide detailed information on their dietary intake and supplement usage to assess nutrient, energy, and other food constituent intakes during two 24-hour dietary recall interviews. The initial dietary recall interview was conducted face-to-face at the MEC, whereas the subsequent one was obtained via telephone 3 to 10 days later. To enhance the accuracy of the estimates, the consumption of α-carotene, β-carotene, β-cryptoxanthin, lutein/zeaxanthin, and lycopene from the diet were averaged across 2 recall periods (the value was utilized instead of the average if only the initial recall were available). However, only lutein/zeaxanthin and lycopene intake from supplements were collected in this specific cycle, and these supplement intakes were also averaged over 2 recalls if available. Consequently, total lutein/zeaxanthin and total lycopene intake were computed by adding both dietary and supplementary sources.

### 2.4. Other covariates

Data on demographics, lifestyle, and health status were obtained through the Computer Assisted Personal Interview system during the household interviews. Participants were classified into different groups, including Mexican American, non-Hispanic White, non-Hispanic Black, non-Hispanic Asian, and other/multiracial, based on the reported race and Hispanic origin information. Education level was categorized into 3 groups: less than high school, high school or equivalent, and college degree or above. The poverty-income ratio (PIR) was categorized as low ( < 1.30), middle (1.30 ≤ PIR < 3.50), and high (≥3.50).^[21]^ We divided physical activity into low ( < 600), moderate (600– < 8000), and high (≥8000) levels by the total weekly minutes metabolic equivalent (MET minute/week) of participants vigorous, moderate work/recreational activity, as well as walking or bicycling.^[22]^ Smoking status was determined by the “Smoking-Cigarette Use” questionnaire into never smokers (having smoked < 100 cigarettes in their lifetime), former smokers (having smoked at least 100 cigarettes in their lifetime but currently quit), and current smokers (having smoked at least 100 cigarettes in their lifetime and still smoking at present). Hypertension was defined by the previous diagnosis, taking prescription medication for hypertension, or an average blood pressure ≥ 140/90 mm Hg.^[23]^ Diabetes mellitus was diagnosed based on either (a) Doctor told you have diabetes or (b) Fasting plasma glucose (FPG) ≥ 7.0 mmol/L or (c) 2-hour PG ≥ 11.1 mmol/L during oral glucose tolerance test or (d) Glycohemoglobin (HbA1c) ≥ 6.5% or (e) Random plasma glucose ≥ 11.1 mmol/L or (f) Use of diabetes medication or insulin.^[24]^ The diagnosis criteria for metabolic syndrome in clinical practice maintained the minor modified version of the Adult Treatment Panel III definition, which has been widely accepted in the United States and other countries.^[25,26]^

Blood samples and physiological examinations were collected following standardized procedures during the participant’s MEC visit. The whole blood and serum specimens underwent processing, storage, and shipping to either the Advanced Research Diagnostics Laboratory at the University of Minnesota or the University of Missouri-Columbia for analysis. In this study, we extracted various laboratory indicators, including alanine aminotransferase (ALT), aspartate aminotransferase (AST), gamma-glutamyl transferase (γ-GT), triglyceride (TG), low-density lipoprotein cholesterol (LDL-C), fasting insulin (FINS), FPG, HbA1c, and high-sensitivity C-reactive protein (Hs-CRP). Further information regarding questionnaire instruments, exam procedure manuals, laboratory methods, and guidelines for dietary recall interviews is accessible on the NHANES website.

### 2.5. Statistical analysis

Following the recommendations provided in the NHANES analytic notes, the dietary day 1 sample weight (WTDRD1) was applied as an appropriate sample weight to this analysis, considering the multi-stage, unequal probability selection design of the NHANES survey. Based on MAFLD status (non-MAFLD vs MAFLD), we summarized the participants’ baseline characteristics. Weighted means with 95% confidence intervals (CIs) were used to present continuous variables, whereas weighted percentages (95% CIs) were used for categorical variables. Weighted linear regression and weighted Chi-square tests were used for continuous and categorical variables to compare the differences between the 2 MAFLD phenotypes in baseline characteristics. The analyses conducted did not use imputation methods for any variable.

Weighted logistic regression models were conducted to explore the association between carotenoid intake and MAFLD. To verify the association and investigate the potential nonlinear relationship, carotenoids, which were continuous, were discretized into tertiles, and the *P* values for trends were computed. Three models were established in this study and were stratified by gender. No adjustments were made to the basic model for any possible confounding variable. Model 1 was adjusted for age, gender, race/ethnicity, educational level, and PIR. In Model 2, additional adjustments were made for body mass index, waist circumference, physical activity, sedentary behavior, smoking status, hypertension, diabetes mellitus, metabolic syndrome, ALT, AST, γ-GT, TG, LDL-C, FINS, FPG, HbA1c, Hs-CRP, and total calories. In sensitivity analyses, we recalculated carotenoid intake by selecting participants with both days of dietary carotenoid intake and using dietary 2-day sample weight (WTDR2D), and then analyzed the association of new carotenoid intake with MAFLD. Furthermore, multiplicative interaction tests were conducted to ascertain whether there was an interaction between gender and carotenoid intake. Restricted cubic splines incorporating 3 knots positioned at the 10th, 50th, and 90th percentiles were employed to flexibly model the association between MAFLD and carotenoid intake.

The R software (https://www.r-project.org/, accessed on March 16 2023, 4.2.3 version, The R Foundation for Statistical Computing, Vienna, Austria) was used for all statistical analyses.

## 3. Results

### 3.1. Baseline characteristics of participants with and without MAFLD

Table 1 displays the baseline characteristics of the study participants categorized by MAFLD status. The weighted prevalence of MAFLD in the entire population of the United States was 51.90%. Of the 2722 participants ultimately enrolled in the study, the overall weighted mean age was 46.31 years, 47.80% were female, and the majority of subjects were non-Hispanic White (weighted percentage: 64.00%). Compared to participants without MAFLD, those with MAFLD were more likely to be older, male, obese, have a larger waist circumference, and have elevated levels of ALT, γ-GT, TG, LDL-C, FINS, FPG, H1Abc, Hs-CRP, and median CAP, as well as a higher incidence of hypertension, metabolic syndrome, and diabetes mellitus. Significant differences in race/ethnicity, educational attainment, physical activity, and smoking status were also observed between the 2 groups, while no significant differences were observed in AST, PIR, and sedentary behavior. The MAFLD group had lower consumption of dietary α-carotene, β-carotene, and total lutein/zeaxanthin than the non-MAFLD group. In contrast, the differences in total calories, dietary β-cryptoxanthin, and total lycopene intake were not statistically significant.

**Table 1:** Baseline characteristics of participants.

Characteristics	Overall(N = 2722)*	Non-MAFLD (N = 1246)*	MAFLD (N = 1476)*	*P* value†
Age (yr)	46.31 (44.85, 47.77)	42.53 (41.07, 43.99)	49.82 (48.06, 51.57)	<.001
Gender, %				<.001
Male	52.20 (46.55, 57.86)	46.89 (43.00, 50.79)	57.12 (53.50, 60.75)	
Female	47.80 (43.99, 51.60)	53.11 (49.21, 57.00)	42.88 (39.25, 46.50)	
Race/ethnicity, %				<.001
Mexican American	8.88 (5.63, 12.14)	5.97 (3.72, 8.23)	11.58 (7.56, 15.59)	
Non-Hispanic White	64.00 (54.60, 73.39)	66.56 (60.08, 73.05)	61.62 (54.23, 69.00)	
Non-Hispanic Black	10.21 (7.69, 12.74)	11.61 (8.51, 14.71)	8.92 (5.43, 12.41)	
Non-Hispanic Asian	5.93 (4.37, 7.49)	6.08 (4.05, 8.12)	5.78 (3.85, 7.71)	
Other/multiracial	10.98 (8.60, 13.37)	9.77 (7.05, 12.49)	12.11 (9.44, 14.77)	
Educational level, %				.005
Less than high school	8.77 (7.49, 10.05)	7.97 (6.18, 9.75)	9.51 (7.88, 11.13)	
High school or equivalent	27.58 (22.76, 32.41)	25.29 (18.91, 31.67)	29.71 (26.12, 33.30)	
College or above	63.65 (57.14, 70.16)	66.74 (59.98, 73.51)	60.78 (56.97, 64.60)	
Poverty-income ratio, %				.260
Low (<1.30)	19.15 (17.00, 21.30)	19.40 (16.72, 22.09)	18.92 (16.48, 21.35)	
Middle (1.30 to < 3.50)	35.43 (29.52, 41.34)	33.88 (28.10, 39.67)	36.86 (30.74, 42.99)	
High (≥3.50)	45.42 (39.91, 50.93)	46.71 (41.29, 52.14)	44.22 (38.13, 50.31)	
Body mass index (kg/m^2^), %				<.001
Underweight or Normal (<25)	28.40 (24.31, 32.49)	52.94 (48.60, 57.27)	5.67 (4.26, 7.08)	
Overweight (25 to < 30)	30.71 (26.98, 34.44)	29.32 (24.97, 33.67)	32.01 (27.45, 36.56)	
Obese (30 or greater)	40.89 (35.59, 46.18)	17.75 (13.05, 22.45)	62.33 (57.64, 67.01)	
Waist circumference (cm)	99.54 (98.20, 100.89)	89.34 (88.11, 90.56)	109.00 (107.58, 110.42)	<.001
Physical activity (MET min/week), %				<.001
Low (<600)	13.06 (10.94, 15.19)	9.19 (6.46, 11.92)	16.65 (13.57, 19.73)	
Moderate (600 to < 8000)	62.27 (57.69, 66.84)	66.62 (62.52, 70.71)	58.24 (53.94, 62.53)	
High (≥8000)	24.67 (20.42, 28.92)	24.19 (20.45, 27.93)	25.11 (21.63, 28.59)	
Sedentary behavior (min/day)	334.64 (319.08, 350.20)	331.51 (310.84, 352.17)	337.55 (318.96, 356.13)	.400
Smoking status, %				<.001
Never smoker	58.72 (54.14, 63.30)	61.29 (57.49, 65.10)	56.34 (52.59, 60.08)	
Former smoker	23.77 (20.65, 26.89)	20.20 (17.33, 23.08)	27.07 (23.36, 30.79)	
Current smoker	17.51 (13.74, 21.28)	18.50 (14.45, 22.56)	16.59 (13.36, 19.82)	
Hypertension, %	35.51 (31.07, 39.96)	30.04 (26.21, 33.87)	69.96 (66.13, 73.79)	<.001
Diabetes mellitus, %	12.33 (10.58, 14.07)	16.46 (11.19, 21.74)	83.54 (78.26, 88.81)	<.001
Metabolic syndrome, %	28.37 (24.48, 32.26)	15.22 (11.11, 19.33)	84.78 (80.67, 88.89)	<.001
Alanine aminotransferase (U/L)	23.96 (22.99, 24.92)	20.29 (18.20, 22.39)	27.28 (26.19, 28.36)	<.001
Aspartate aminotransferase (U/L)	22.87 (22.03, 23.71)	22.33 (20.27, 24.38)	23.36 (22.54, 24.19)	.060
Gamma-glutamyl transferase (IU/L)	29.40 (27.87, 30.93)	23.41 (21.22, 25.60)	34.83 (31.64, 38.03)	<.001
Triglyceride (mmol/L)	1.28 (1.18, 1.38)	0.95 (0.88, 1.01)	1.57 (1.40, 1.74)	<.001
LDL-cholesterol (mmol/L)	2.89 (2.82, 2.97)	2.81 (2.71, 2.91)	2.97 (2.80, 3.14)	.002
Fasting insulin (uU/ml)	12.35 (11.14, 13.55)	7.78 (6.99, 8.57)	16.32 (14.53, 18.10)	<.001
Fasting plasma glucose (mmol/L)	6.05 (5.93, 6.17)	5.52 (5.45, 5.59)	6.51 (6.29, 6.73)	<.001
Glycohemoglobin (%)	5.60 (5.56, 5.64)	5.35 (5.31, 5.39)	5.82 (5.75, 5.90)	<.001
Hs-CRP (mg/L)	3.57 (3.13, 4.02)	2.35 (1.96, 2.75)	4.68 (4.14, 5.22)	<.001
Total calories (kcal/d)	2133.97 (2081.82, 2186.12)	2100.02 (2025.95, 2174.10)	2165.42 (2118.93, 2211.91)	.060
Dietary α-carotene (mg/d)	0.39 (0.33, 0.45)	0.44 (0.37, 0.52)	0.34 (0.29, 0.40)	.002
Dietary β-carotene (mg/d)	2.47 (2.19, 2.76)	2.86 (2.43, 3.28)	2.12 (1.89, 2.35)	<.001
Dietary β-cryptoxanthin (mg/d)	0.09 (0.08, 0.11)	0.09 (0.08, 0.11)	0.09 (0.07, 0.12)	.940
Total Lutein/zeaxanthin (mg/d)	1.90 (1.68, 2.11)	2.26 (1.85, 2.67)	1.56 (1.43, 1.69)	<.001
Total Lycopene (mg/d)	5.05 (4.53, 5.57)	5.26 (4.62, 5.90)	4.85 (4.21, 5.49)	.130
CAP (dB/m)	260.84 (256.65, 265.03)	209.38 (206.35, 212.41)	308.53 (305.42, 311.63)	<.001

CAP = controlled attenuation parameter, Hs-CRP = high-sensitivity C-reactive, MAFLD = metabolic dysfunction-associated fatty liver diseaseprotein.

* Weighted means or weighted percentages with their 95% confidence intervals (CIs) for continuous or categorical variables.

† The *P* values were calculated by weighted linear regression model or weighted Chi-squared test for continuous or categorical variables.

### 3.2. Association of carotenoid intake with MAFLD

**Figure 2.**
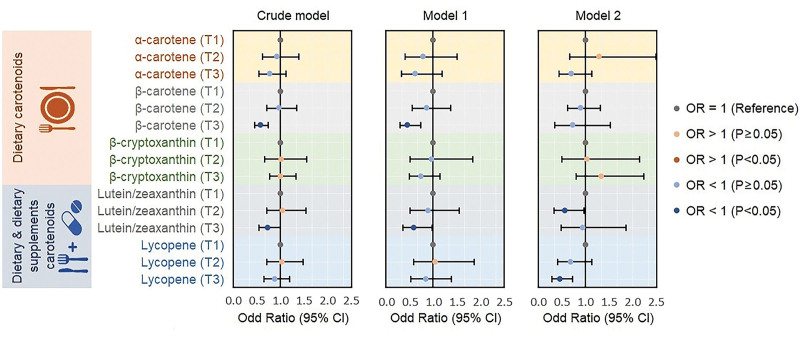
Weighted logistic regression analysis models showing the association between MAFLD risk and carotenoid intake. Crude model: Unadjusted model. Model 1: Adjusted for age, gender, race/ethnicity, educational level, and poverty-income ratio. Model 2: Additionally adjusted for body mass index, waist circumference, physical activity, sedentary behavior, smoking status, hypertension, diabetes mellitus, metabolic syndrome, ALT, AST, γ-GT, TG, LDL-C, FINS, FPG, HbA1c, Hs-CRP, and total calories. γ-GT = gamma-glutamyl transferase, ALT = alanine aminotransferase, AST = aspartate aminotransferase, FINS = fasting insulin, FPG = fasting plasma glucose, HbA1c = glycohemoglobin, Hs-CRP = high-sensitivity C-reactive protein, LDL-C = low-density lipoprotein cholesterol, MAFLD = metabolic dysfunction-associated fatty liver disease, OR = odd ratio, TG = triglyceride.

As illustrated in Figure 2, weighted logistic regression analysis demonstrated the association between carotenoid intake and MAFLD. In the unadjusted model, the highest tertile of dietary β-carotene and total lutein/zeaxanthin was inversely correlated with MAFLD compared with the lowest tertile (β-carotene _T3 vs T1_: OR = 0.579, 95% CI: 0.450–0.744, *P* < .001; lutein/zeaxanthin _T3 vs T1_: OR = 0.736, 95% CI: 0.546–0.993, *P* = .045). Model 1 showed comparable findings after adjusting for sociodemographic characteristics. However, no significant association was observed between dietary β-carotene and MAFLD after full adjustment (Model 2), and the notable negative correlation between the intake of total lutein/zeaxanthin and MAFLD shifted from the highest tertile to the second tertile (lutein/zeaxanthin _T2 vs T1_: OR = 0.568, 95% CI: 0.333–0.969, *P* = .039). Furthermore, the risk of MAFLD was obviously reduced in the top tertile of lycopene intake compared to that in the lowest tertile (lycopene _T3 vs T1_: OR = 0.459, 95% CI: 0.289–0.729, *P* = .003). In sensitivity analysis, roughly consistent results were observed (see Table S1, Supplemental Digital Content, http://links.lww.com/MD/L81, which illustrates the association between carotenoid intake and MAFLD in sensitivity analysis).

### 3.3. Association between carotenoid intake and MAFLD stratified by gender

Table 2 exhibits the correlation between carotenoid intake and MAFLD in different gender subgroups determined by weighted logistic regression. Significant inverse correlations were found in the crude model between MAFLD and the highest tertile of dietary β-carotene, total lutein/zeaxanthin, and total lycopene consumption in the female group, with all of the above linear trends being statistically significant. Nevertheless, the negative association in females only existed in total lycopene intake after adjusting for sociodemographic characteristics, anthropometric indices, standard biochemical indexes, and comorbidities, and the linear pattern was still significant (*P* for trend = .027). Regarding the interaction analysis of gender on carotenoid intake, there was an interaction of gender on total lycopene intake in the basic model (*P* for interaction = .014), which vanished after accounting for confounding factors (*P* for interaction = .299). Consistent results were also obtained from the sensitivity analysis (see Table S2, Supplemental Digital Content, http://links.lww.com/MD/L82, which demonstrates the association between carotenoid intake and MAFLD in each gender in sensitivity analysis).

**Table 2:** Odd ratio estimates for the association between the carotenoid intake and metabolic dysfunction-associated fatty liver disease in each gender.

Carotenoids	T1	T2 (OR 95%CI)	*P* value	T3 (OR 95%CI)	*P* value	*P* for trend	*P* for interaction
α-carotene							
Unadjusted							.079
Male	1.000	0.856 (0.522,1.404)	.510	0.997 (0.636,1.564)	.989	.944	
Female	1.000	1.019 (0.613,1.695)	.938	0.640 (0.394,1.039)	.068	.068	
Adjusted*							.810
Male	1.000	1.031 (0.349, 3.045)	.953	0.780 (0.329, 1.851)	.549	.533	
Female	1.000	1.572 (0.689, 3.589)	.261	0.725 (0.402, 1.309)	.264	.381	
β-carotene							
Unadjusted							.028
Male	1.000	1.094 (0.733,1.631)	.637	0.813 (0.560,1.182)	.254	.261	
Female	1.000	0.857 (0.553,1.327)	.459	0.416 (0.283,0.611)	<.001	<.001	
Adjusted							.294
Male	1.000	0.631 (0.314, 1.268)	.180	0.704 (0.270, 1.835)	.446	.518	
Female	1.000	1.409 (0.819, 2.425)	.198	0.704 (0.357, 1.388)	.288	.308	
β-cryptoxanthin							
Unadjusted							.671
Male	1.000	1.111 (0.638,1.935)	.690	1.053 (0.717,1.547)	.775	.778	
Female	1.000	0.869 (0.540,1.399)	.536	0.889 (0.565,1.397)	.583	.557	
Adjusted							.870
Male	1.000	1.057 (0.469, 2.383)	.885	1.128 (0.526, 2.420)	.741	.743	
Female	1.000	1.025 (0.425, 2.470)	.954	1.546 (0.600, 3.984)	.343	.364	
Lutein/zeaxanthin							
Unadjusted							.020
Male	1.000	1.096 (0.665,1.808)	.697	1.036 (0.707,1.519)	.844	.848	
Female	1.000	0.961 (0.610,1.515)	.853	0.510 (0.310,0.837)	.012	.013	
Adjusted							.900
Male	1.000	0.583 (0.311, 1.095)	.088	1.011 (0.409, 2.499)	.980	.861	
Female	1.000	0.543 (0.228, 1.294)	.155	0.913 (0.378, 2.209)	.830	.773	
Lycopene							
Unadjusted							.014
Male	1.000	1.086 (0.619,1.905)	.756	1.128 (0.752,1.693)	.532	.527	
Female	1.000	0.975 (0.676,1.405)	.884	0.563 (0.416,0.760)	.001	.002	
Adjusted							.299
Male	1.000	0.709 (0.307, 1.637)	.395	0.634 (0.302, 1.331)	.210	.213	
Female	1.000	0.614 (0.237, 1.592)	.293	0.309 (0.117, 0.820)	.022	.027	

CI = confidence Interval, OR = odd ratio.

* Adjusted Model: Adjusted for age, race/ethnicity, educational level, poverty-income ratio, body mass index, waist circumference, physical activity, sedentary behavior, smoking status, hypertension, diabetes mellitus, metabolic syndrome, ALT, AST, γ-GT, TG, LDL-C, FINS, FPG, HbA1c, Hs-CRP, and total calories.

### 3.4. Analysis of restricted cubic spline regression

**Figure 3.**
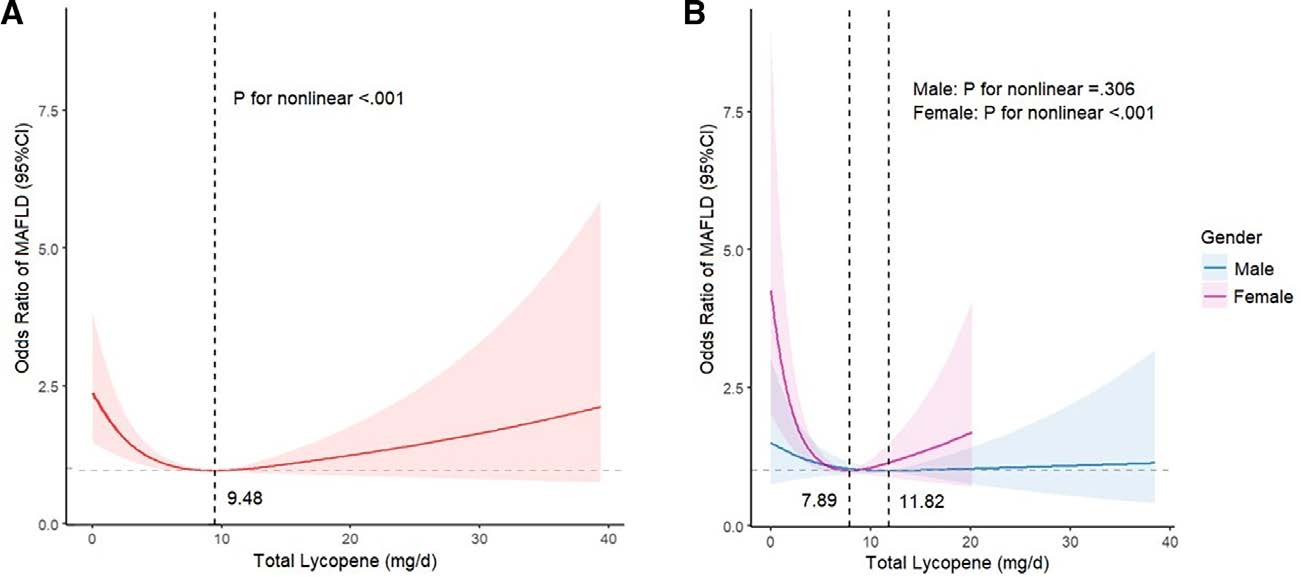
Restricted cubic spline depicts the association between total lycopene intake and MAFLD: (A) Restricted cubic spline analysis among all survey subjects, (B) restricted cubic spline analysis across gender subgroups. The red, blue, and purple lines represent ORs, and the red, blue, and purple transparent areas represent 95% CIs. Restricted cubic spline models are adjusted for age, gender, race/ethnicity, educational level, poverty-income ratio, body mass index, waist circumference, physical activity, sedentary behavior, smoking status, hypertension, diabetes mellitus, metabolic syndrome, ALT, AST, γ-GT, TG, LDL-C, FINS, FPG, HbA1c, Hs-CRP, and total calories. γ-GT = gamma-glutamyl transferase, ALT = alanine aminotransferase, AST = aspartate aminotransferase, CI = confidence interval, FINS = fasting insulin, FPG = fasting plasma glucose, HbA1c = glycohemoglobin, Hs-CRP = high-sensitivity C-reactive protein, LDL-C = low-density lipoprotein cholesterol, MAFLD = metabolic dysfunction-associated fatty liver disease, TG = triglyceride.

**Figure 4.**
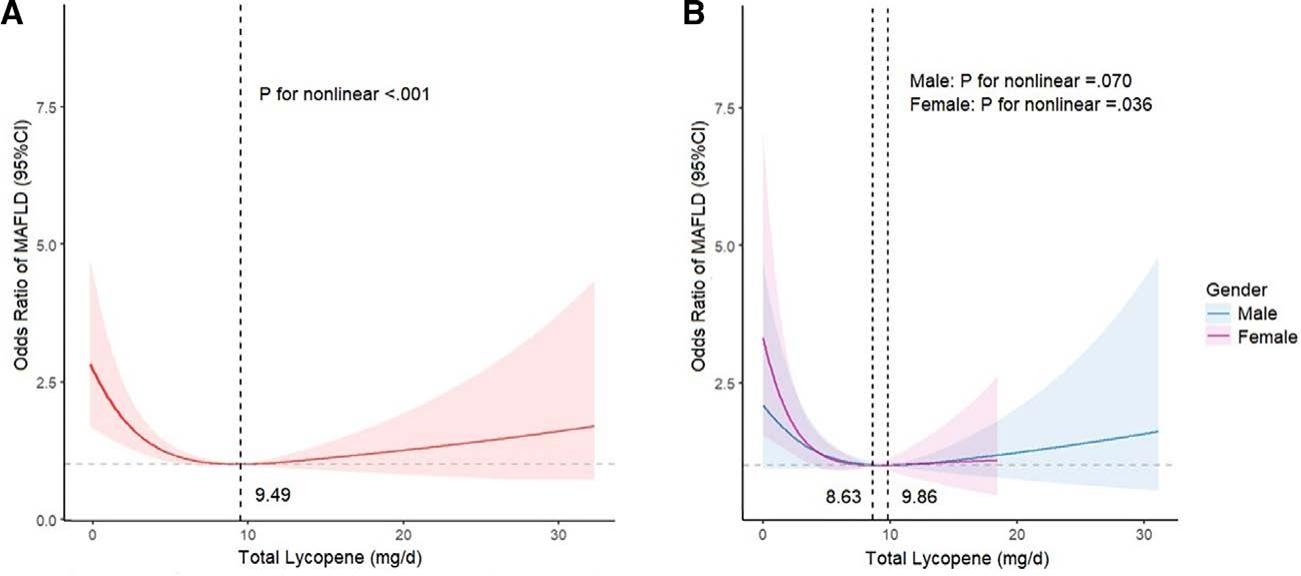
Restricted cubic spline demonstrates the association between total lycopene intake and MAFLD in sensitivity analysis: (A) Restricted cubic spline analysis among all survey subjects, (B) restricted cubic spline analysis across gender subgroups. The red, blue, and purple lines represent ORs, and the red, blue, and purple transparent areas represent 95% CIs. Restricted cubic spline models are adjusted for age, gender, race/ethnicity, educational level, poverty-income ratio, body mass index, waist circumference, physical activity, sedentary behavior, smoking status, hypertension, diabetes mellitus, metabolic syndrome, ALT, AST, γ-GT, TG, LDL-C4, FINS, FPG, HbA1c, Hs-CRP, and total calories. γ-GT = gamma-glutamyl transferase, ALT = alanine aminotransferase, AST = aspartate aminotransferase, CI = confidence interval, FINS = fasting insulin, FPG = fasting plasma glucose, HbA1c = glycohemoglobin, Hs-CRP = high-sensitivity C-reactive protein, LDL-C = low-density lipoprotein cholesterol, MAFLD = metabolic dysfunction-associated fatty liver disease, TG = triglyceride.

In restricted cubic spline regression analysis, we identified a noteworthy nonlinear association between the total intake of lycopene and MAFLD (*P* for nonlinear < .001, Fig. 3A) after adjusting for various covariates. Given the prominent U-shaped association between total lycopene intake and MAFLD risk, Figure 3A demonstrates a considerable risk reduction within the lower range of total lycopene intake, which reached the lowest risk around 9.48 mg/day and then increased thereafter. In the gender subgroup analysis, a notable nonlinear association between lycopene consumption and MAFLD persisted among females (*P* for nonlinear < .001, Fig. 3B) but not in males (*P* for nonlinear = .306, Fig. 3B). Despite slight differences in the results of sensitivity analysis, the nonlinear relationship remained consistent (Fig. 4).

## 4. Discussion

To elucidate the potential effect of carotenoid intake on the risk of MAFLD, a cross-sectional analysis was performed on 2722 participants from the NHANES 2017 to 2018. In this study, we demonstrated that intake of β-carotene, lutein/zeaxanthin, and lycopene may have protective effects against MAFLD. The restricted cubic spline analysis revealed that the aforementioned associations were primarily linear, whereas a nonlinear relationship between lycopene and MAFLD was detected, which manifested predominantly in females.

The multiple parallel hits hypothesis suggests that significant hepatic lipid accumulation and insulin resistance cause various metabolic alterations and lipotoxins production, which can lead to mitochondrial dysfunction, endoplasmic reticulum stress, and excessive reactive oxygen species generation, ultimately promoting the progression of MAFLD.^[27,28]^ Several studies have shown that vitamin supplementation may protect liver tissue by reducing insulin resistance, lipid peroxidation, and fatty acid synthesis, and improving hepatic steatosis.^[29,30]^ Carotenoids, the precursors of vitamin A, can regulate gene expression and immune responses and have potent antioxidant properties, suggesting a potential protective role in MAFLD.^[31]^ In our study, no statistically significant correlation was found between the intake of α-carotene, β-cryptoxanthin, and MAFLD, despite earlier research indicating their potential in preventing NAFLD.^[11,32]^ One possible explanation for this inconsistency is that past studies had a limited number of participants.

Our results showed that the association between β-carotene and MAFLD was relatively stable. Despite the lack of direct evidence, several research results support our findings. As a powerful anti-inflammatory and antioxidant micronutrient, β-carotene has shown promise in ameliorating hepatic steatosis and mitigating inflammation in individuals with NAFLD.^[33]^ A mediation analysis study using data from the NHANES 2007 to 2010 revealed that dietary β-carotene may decrease the likelihood of NAFLD by suppressing inflammation.^[34]^ Another case-control study, which recruited 24 control participants and 62 biopsy-proven NAFLD patients, revealed a notable decrease in serum β-carotene levels among NAFLD patients compared to the control group.^[35]^ Furthermore, serum β-carotene levels gradually decreased with increasing histological severity of NAFLD, implying a potential protective effect of β-carotene on liver pathology.^[35]^ Interestingly, Liu et al^[12]^ discovered that a greater consumption of β-carotene was associated with a decrease in hepatic steatosis, although the relationship might be nonlinear. In contrast, our study suggested a potential linear relationship between β-carotene and MAFLD, which may be attributed to variations in the target study groups.

Lutein/zeaxanthin is mainly concentrated in the macula of the retina but is also distributed in the liver, adipose tissue, and brain, where it exerts potent antioxidant effects by neutralizing reactive oxygen species and directly quenching free radicals.^[36]^ As shown in Figure 2, we have suggested the protective benefits of lutein/zeaxanthin intake on MAFLD. Similarly, previous research has indicated a negative correlation between the occurrence of NAFLD and plasma lutein/zeaxanthin in middle-aged and elderly Chinese populations, even after adjusting for potential confounders (all *P* values < .001).^[37]^ Lutein supplementation has been demonstrated to alleviate hepatic lipid accumulation and improve insulin resistance in NAFLD rats induced by a high-fat diet.^[38]^ In animal models fed a zeaxanthin-supplemented diet for 6 weeks, it was also observed that zeaxanthin treatment remarkably reduced hepatic lipid peroxidation and attenuated oxidative stress in a dose-dependent manner.^[39]^ The underlying mechanism may be ascribed to the upregulation of proteins that are involved in lipid metabolism and insulin signal transduction, such as Sirtuin 1, peroxisome proliferators activated receptor-α, phosphatidylinositol 3-kinase, and glucose transporter-2, which is similar to the potential mechanism of vitamin K on MAFLD.^[40]^

As for lycopene, previous studies have yielded inconsistent findings concerning its effects. Numerous prior studies in animals and humans have suggested that it could prevent NAFLD by ameliorating hepatic steatosis and attenuating inflammation through its antioxidant properties.^[6,13,41]^ Likewise, a recent meta-analysis concluded that higher lycopene levels are linked to reduced metabolic diseases such as NAFLD, type 2 diabetes mellitus, dyslipidemia, and metabolic syndrome.^[42]^ However, a cohort study conducted in Japan found no significant relationship between NAFLD and serum lycopene levels.^[43]^ What’s even more intriguing is that a Mendelian randomization study conducted by Chen et al^[44]^ discovered that higher consumption of lycopene may lead to an elevated risk of NAFLD. However, it is remarkable that the restricted cubic spline analysis we performed showed evidence of a nonlinear relationship between total lycopene intake and MAFLD, which was particularly significant in females. As mentioned in a systemic review, the recommendations for dietary lycopene intake may be uncertain due to interindividual response variations in age, gender, disease state, and genetic makeup.^[45]^ And differential effects of female hormones, such as estrogen and progesterone, on liver metabolism may explain the observed gender differences.^[46,47]^ Future studies with appropriate consideration of hormonal status, gender, and sociocultural gender differences are warranted and may help us to comprehensively understand the risk, mechanisms, and therapeutic targets of MAFLD.

To the best of our knowledge, this study is the first to investigate the association between carotenoids and MAFLD. Our study has several advantages, including strict protocols and quality assurance measures, a nationally representative sample, and NHANES data with excellent quality. Additionally, we found potential protective effects of β-carotene, lutein/zeaxanthin, and lycopene against MAFLD, along with the potential existence of a nonlinear correlation between lycopene and MAFLD. However, our study has several limitations. First, the consumption of carotenoids was evaluated through two 24-hour dietary recall periods, which may not accurately mirror the true daily intake. Second, evidence of hepatic steatosis in MAFLD was determined using elastography rather than liver biopsy because liver biopsy is impractical in large-scale population studies. Moreover, despite our study being controlled for multiple covariates, the extent of their adjustment and residual confounding from unmeasured factors still require attention in observational studies. Finally, given that this cross-sectional study design may be subject to memory bias and is unable to establish cause-and-effect relationships, it is necessary to validate our findings through future studies that follow a prospective cohort design.

## 5. Conclusion

In summary, our findings suggested that an increased intake of β-carotene, lutein/zeaxanthin, and lycopene may reduce the risk of MAFLD. The association between MAFLD and total lycopene intake was nonlinear, especially in females. In the future, the causal relationship and precise mechanism between carotenoids and MAFLD should be further validated in large-scale prospective studies.

## Acknowledgements

The authors thank all NHANES participants and staff for their valuable efforts and contributions. We also thank Zhang Jing (Second Department of Infectious Disease, Shanghai Fifth People’s Hospital, Fudan University) for his work on the NHANES database. His outstanding work, nhanesR package and webpage, makes it easier for us to explore the NHANES database.

## Authors contributions

**Conceptualization:** Hang Zhang, Li Li.

**Data curation:** Hang Zhang, Li Li, Lei Jia.

**Formal analysis:** Hang Zhang, Lei Jia.

**Investigation:** Hang Zhang, Jinchun Liu.

**Methodology:** Hang Zhang, Li Li, Jinchun Liu.

**Software:** Hang Zhang, Li Li.

**Validation:** Hang Zhang, Jinchun Liu.

**Visualization:** Hang Zhang, Lei Jia.

**Writing – original draft:** Hang Zhang.

**Writing – review & editing:** Li Li, Jinchun Liu.

## Supplementary Material




