# Supplementary material for: Association between carotenoid intake and metabolic dysfunction-associated fatty liver disease among US adults: A cross-sectional study
Source: Medicine (Baltimore). 2023 Dec 22;102(51):e36658. doi: 10.1097/MD.0000000000036658 (PMC10735096; doi:10.1097/MD.0000000000036658)
Supplement: Supplementary file 1 [file medi-102-e36658-s001.docx]

**Supplemental Digital Content**

**Article Title:**

**Association between carotenoid intake and metabolic dysfunction-associated fatty liver disease among US adults: a cross-sectional study.**

**First Author:**

**Hang Zhang**

**Table 1.** Weighted logistic regression analysis models showing the association between carotenoid intake and metabolic dysfunction-associated fatty liver disease in sensitivity analysis.

| **Carotenoids** | **Crude model** | | | **Model 1** | | | **Model 2** | | |
| --- | --- | --- | --- | --- | --- | --- | --- | --- | --- |
|  | **OR (95% CI)** | ***p*** | ***p* for trend** | **OR (95% CI)** | ***p*** | ***p* for trend** | **OR (95% CI)** | ***p*** | ***p* for trend** |
| α-carotene |  |  | .130 |  |  | .076 |  |  | .145 |
| T1 | Ref | Ref |  | Ref | Ref |  | Ref | Ref |  |
| T2 | 0.807 (0.503, 1.295) | .346 |  | 0.675 (0.312, 1.457) | .202 |  | 1.170 (0.552, 2.480) | .662 |  |
| T3 | 0.710 (0.480, 1.050) | .081 |  | 0.544 (0.260, 1.136) | .078 |  | 0.687 (0.417, 1.132) | .130 |  |
| β-carotene |  |  | **.001** |  |  | **.012** |  |  | .744 |
| T1 | Ref | Ref |  | Ref | Ref |  | Ref | Ref |  |
| T2 | 0.933 (0.636, 1.369) | .704 |  | 0.807 (0.437, 1.490) | .346 |  | 0.912 (0.573, 1.449) | .676 |  |
| T3 | 0.578 (0.414, 0.808) | **.004** |  | 0.458 (0.245, 0.859) | **.029** |  | 0.874 (0.371, 2.057) | .742 |  |
| β-cryptoxanthin |  |  | .979 |  |  | .102 |  |  | .413 |
| T1 | Ref | Ref |  | Ref | Ref |  | Ref | Ref |  |
| T2 | 1.020 (0.652, 1.598) | .924 |  | 0.974 (0.474, 1.999) | .913 |  | 1.012 (0.541, 1.893) | .968 |  |
| T3 | 1.009 (0.748, 1.361) | .950 |  | 0.756 (0.472, 1.211) | .155 |  | 1.280 (0.679, 2.412) | .420 |  |
| Lutein/zeaxanthin |  |  | **.026** |  |  | **.043** |  |  | .471 |
| T1 | Ref | Ref |  | Ref | Ref |  | Ref | Ref |  |
| T2 | 1.111 (0.760, 1.624) | .559 |  | 0.954 (0.530, 1.718) | .816 |  | 0.741 (0.402, 1.366) | .313 |  |
| T3 | 0.771 (0.598, 0.995) | **.046** |  | 0.647 (0.399, 1.050) | .065 |  | 1.291 (0.629, 2.651) | .460 |  |
| Lycopene |  |  | .218 |  |  | .204 |  |  | **.004** |
| T1 | Ref | Ref |  | Ref | Ref |  | Ref | Ref |  |
| T2 | 1.085 (0.672, 1.752) | .719 |  | 1.099 (0.531, 2.278) | .706 |  | 0.515 (0.317, 0.836) | **.011** |  |
| T3 | 0.868 (0.607, 1.243) | .411 |  | 0.816 (0.453, 1.472) | .354 |  | 0.393 (0.218, 0.706) | **.004** |  |

Crude model: Unadjusted model.

Model 1: Adjusted for age, gender, race/ethnicity, educational level, and poverty-income ratio.

Model 2: Additionally adjusted for body mass index, waist circumference, physical activity, sedentary behavior, smoking status, hypertension, diabetes mellitus, metabolic syndrome, ALT, AST, γ-GT, TG, LDL-C, FINS, FPG, HbA1c, Hs-CRP, and total calories.

Abbreviations: OR, Odd Ratio; Ref, reference.

Statistical significance was determined with a two-tailed p < .05.
