# Supplementary material for: Association between carotenoid intake and metabolic dysfunction-associated fatty liver disease among US adults: A cross-sectional study
Source: Medicine (Baltimore). 2023 Dec 22;102(51):e36658. doi: 10.1097/MD.0000000000036658 (PMC10735096; doi:10.1097/MD.0000000000036658)
Supplement: Supplementary file 2 [file medi-102-e36658-s002.docx]

**Supplemental Digital Content**

**Article Title:**

**Association between carotenoid intake and metabolic dysfunction-associated fatty liver disease among US adults: a cross-sectional study.**

**First Author:**

**Hang Zhang**

**Table 2.** Odd Ratio estimates for the association between the carotenoid intake and metabolic dysfunction-associated fatty liver disease in each gender in sensitivity analysis.

| **Carotenoids** | **T1** | **T2 (OR 95%CI)** | ***p*** | **T3 (OR 95%CI)** | ***p*** | ***p* for trend** | ***p* for**  **interaction** |
| --- | --- | --- | --- | --- | --- | --- | --- |
| α-carotene |  |  |  |  |  |  |  |
| Unadjusted |  |  |  |  |  |  | .014 |
| Male | 1.000 | 0.760 (0.425, 1.359) | .326 | 1.020 (0.652, 1.595) | .925 | .976 |  |
| Female | 1.000 | 0.874 (0.508, 1.502) | .600 | 0.532 (0.307, 0.922) | .028 | .027 |  |
| Adjusted * |  |  |  |  |  |  | .731 |
| Male | 1.000 | 1.115 (0.442, 2.814) | .805 | 1.291 (0.608, 2.742) | .480 | .483 |  |
| Female | 1.000 | 1.327 (0.614, 2.866) | .446 | 0.538 (0.279, 1.037) | .062 | .104 |  |
| β-carotene |  |  |  |  |  |  |  |
| Unadjusted |  |  |  |  |  |  | **.010** |
| Male | 1.000 | 1.132 (0.720, 1.779) | .563 | 0.942 (0.603, 1.471) | .777 | .808 |  |
| Female | 1.000 | 0.750 (0.402, 1.399) | .337 | 0.357 (0.216, 0.591) | **<.001** | **<.001** |  |
| Adjusted |  |  |  |  |  |  | .734 |
| Male | 1.000 | 0.805 (0.426, 1.520) | .478 | 1.413 (0.427, 4.677) | .548 | .536 |  |
| Female | 1.000 | 0.998 (0.518, 1.922) | .994 | 0.557 (0.234, 1.323) | .170 | .175 |  |
| β-cryptoxanthin |  |  |  |  |  |  |  |
| Unadjusted |  |  |  |  |  |  | .757 |
| Male | 1.000 | 1.051 (0.561, 1.967) | .868 | 0.950 (0.634, 1.423) | .787 | .779 |  |
| Female | 1.000 | 0.915 (0.597, 1.402) | .659 | 1.009 (0.648, 1.572) | .965 | .999 |  |
| Adjusted |  |  |  |  |  |  | .501 |
| Male | 1.000 | 0.863 (0.332, 2.247) | .747 | 0.929 (0.354, 2.436) | .873 | .899 |  |
| Female | 1.000 | 1.201 (0.590, 2.445) | .590 | 1.970 (0.652, 5.955) | .211 | .213 |  |
| Lutein/zeaxanthin |  |  |  |  |  |  |  |
| Unadjusted |  |  |  |  |  |  | **.007** |
| Male | 1.000 | 1.266 (0.793, 2.020) | .296 | 1.207 (0.802, 1.816) | .339 | .332 |  |
| Female | 1.000 | 0.947 (0.554, 1.620) | .831 | 0.495 (0.325, 0.754) | **.003** | **.003** |  |
| Adjusted |  |  |  |  |  |  | .966 |
| Male | 1.000 | 0.897 (0.364, 2.208) | .800 | 1.904 (0.646, 5.606) | .223 | .234 |  |
| Female | 1.000 | 0.682 (0.273, 1.704) | .387 | 1.030 (0.399, 2.661) | .948 | .977 |  |
| Lycopene |  |  |  |  |  |  |  |
| Unadjusted |  |  |  |  |  |  | **.031** |
| Male | 1.000 | 1.248 (0.637, 2.448) | .489 | 1.190 (0.748, 1.894) | .432 | .449 |  |
| Female | 1.000 | 0.948 (0.550, 1.631) | .834 | 0.528 (0.334, 0.836) | **.010** | **.014** |  |
| Adjusted |  |  |  |  |  |  | .820 |
| Male | 1.000 | 0.472 (0.177, 1.263) | .125 | 0.476 (0.197, 1.146) | .092 | .098 |  |
| Female | 1.000 | 0.544 (0.239, 1.236) | .135 | 0.361 (0.146, 0.891) | **.030** | **.032** |  |

* Adjusted Model: Adjusted for age, race/ethnicity, educational level, poverty-income ratio, body mass index, waist circumference, physical activity, sedentary behavior, smoking status, hypertension, diabetes mellitus, metabolic syndrome, ALT, AST, γ-GT, TG, LDL-C, FINS, FPG, HbA1c, Hs-CRP, and total calories.

Abbreviations: OR, Odd Ratio; CI, Confidence Interval.
